# Pharmacokinetic and Toxicodynamic Characterization of a Novel Doxorubicin Derivative

**DOI:** 10.3390/pharmaceutics9030035

**Published:** 2017-09-13

**Authors:** Samaa Alrushaid, Casey L. Sayre, Jaime A. Yáñez, M. Laird Forrest, Sanjeewa N. Senadheera, Frank J. Burczynski, Raimar Löbenberg, Neal M. Davies

**Affiliations:** 1 College of Pharmacy, Rady Faculty of Health Sciences, University of Manitoba, Winnipeg, MB R3E 0T5, Canada; umalrush@myumanitoba.ca (S.A.); csayre@roseman.edu (C.L.S.); Frank.Burczynski@umanitoba.ca (F.J.B.); 2 College of Pharmacy, Roseman University of Health Sciences, South Jordan, UT 84096, USA; 3 YARI International Group, New Brunswick, NJ 08901 and INDETEC Corp., Lima, Peru; jaimeyanez@gmail.com; 4 Department of Pharmaceutical Chemistry, School of Pharmacy, University of Kansas, Lawrence, KS 66047, USA; mforrest@ku.edu (M.L.F.); nilendrasns@yahoo.com (S.N.S.); 5 Faculty of Pharmacy and Pharmaceutical Sciences, University of Alberta, Edmonton, AB T6G 2R3, Canada; raimar@ualberta.ca

**Keywords:** doxorubicin, quercetin, pharmacokinetics, bioavailability, lymphatics transport, toxicity

## Abstract

Doxorubicin (Dox) is an effective anti-cancer medication with poor oral bioavailability and systemic toxicities. DoxQ was developed by conjugating Dox to the lymphatically absorbed antioxidant quercetin to improve Dox’s bioavailability and tolerability. The purpose of this study was to characterize the pharmacokinetics and safety of Dox after intravenous (IV) and oral (PO) administration of DoxQ or Dox (10 mg/kg) and investigate the intestinal lymphatic delivery of Dox after PO DoxQ administration in male Sprague–Dawley rats. Drug concentrations in serum, urine, and lymph were quantified by HPLC with fluorescence detection. DoxQ intact IV showed a 5-fold increase in the area under the curve (AUC)—18.6 ± 1.98 compared to 3.97 ± 0.71 μg * h/mL after Dox—and a significant reduction in the volume of distribution (V_ss_): 0.138 ± 0.015 versus 6.35 ± 1.06 L/kg. The fraction excreted unchanged in urine (f_e_) of IV DoxQ and Dox was ~5% and ~11%, respectively. Cumulative amounts of Dox in the mesenteric lymph fluid after oral DoxQ were twice as high as Dox in a mesenteric lymph duct cannulation rat model. Oral DoxQ increased AUC of Dox by ~1.5-fold compared to after oral Dox. Concentrations of β-N-Acetylglucosaminidase (NAG) but not cardiac troponin (cTnI) were lower after IV DoxQ than Dox. DoxQ altered the pharmacokinetic disposition of Dox, improved its renal safety and oral bioavailability, and is in part transported through intestinal lymphatics.

## 1. Introduction

Doxorubicin (Dox) is an effective anti-cancer medication that has been clinically used to treat a variety of cancers including breast, ovarian, and lymphoma [1,2,3,4,5]. Despite the clinical effectiveness of Dox, its use is limited by off-target adverse effects, particularly dose-related cardiotoxicity and renal toxicity, which involve free radical formation and tissue damage. Dox formulations that are pegylated and in liposomes are utilized in medications, including Doxil™ and Caelyx™ [6]. Pegylated (polyethylene glycol coated) liposome-encapsulated forms of Dox result in an increased concentration of Dox in the skin and a side effect called palmar plantar erythrodysesthesia or hand–foot syndrome [7]. Non-pegylated liposomal Dox called Myocet™ does not have a polyethylene glycol coating, and therefore does not result in the same rate of hand–foot syndrome. This liposomal encapsulation of Dox limits but does not eliminate the cardiotoxic effects of the drug. This damage is caused by the generation of reactive oxidative species (ROS) such as superoxide and hydrogen peroxide upon the reduction of Dox to form electron-deficient semiquinone [8]. Various additional drug delivery approaches have been undertaken to overcome the toxicity limitations of Dox, such as utilization of micelles [9], synthetic polymer conjugates [10], and antibody targeted carriers [11], with varied degrees of success. We have previously demonstrated that hyaluronan, a biopolymeric nanocarrier, improves survival and reduces the toxicity of Dox in xenografts of human breast cancer through the localization of Dox into the lymphatics [8].

Dox is a substrate of both the P–glycoprotein (P–gp) efflux pump [12] and cytochrome P450 metabolic enzymes [13], both of which contribute to its overall disposition, poor oral absorption, and low oral bioavailability. For this reason, Dox is only currently available as a parenteral treatment administered intravenously. We have previously reported the synthesis of a Dox-quercetin derivative designed to overcome P–gp efflux and CYP inhibition [14] as quercetin is a natural flavonoid that exhibits inhibitory effects on CYP3A4 and P–gp [15] and an antioxidant that scavenges free radicals. Our in vitro investigation of DoxQ [14] revealed that both Dox and quercetin are released from the conjugate over time. Furthermore, DoxQ inhibited CYP3A4, a major metabolic enzyme involved in the first pass effect, and demonstrated higher cellular uptake by P–gp-positive (MDCK–MDR) cells compared to free Dox. The inhibitory effects of DoxQ on CYP3A4 and P–gp may improve the oral absorption and bioavailability of Dox in vivo. Additionally, DoxQ retained anti-cancer activity in a triple negative murine breast cancer cell line and was less toxic to both rat and human cardiomyocytes. The cardioprotective mechanism of DoxQ involved scavenging ROS, suppression of oxidative stress, and cardiac hypertrophy markers, and also inhibitory effects on CYP1B1, all of which contribute to Dox’s induced cardiotoxicity. Taken together, the in vitro results of DoxQ showed promise at mitigating the cardiotoxicity of Dox and may also mitigate its poor oral bioavailability in vivo by inhibiting CYP3A4 and P–gp [14]. The antioxidant effects of DoxQ may also mitigate the renal toxicity induced by Dox and improve its overall tolerability in vivo.

In addition to quercetin’s antioxidant activity and inhibitory effects on CYP3A4 and P–gp, it is naturally absorbed into intestinal lymphatics after gastric or intraduodenal administration [16,17,18]; this property may be utilized as a novel strategy to deliver Dox into lymphatics. Following oral administration, molecules and drugs are either absorbed from the intestinal mucosa into the blood stream via the hepatic portal vein or into lymphatics via the intestinal lymphatic pathway. Most small molecules and drugs administered orally enter systemic circulation via blood capillaries and become subject to hepatic metabolism before entering the vasculature. In contrast, highly lipophilic molecules and macromolecules such as proteins associate with chylomicrons in the intestinal mucosa and enter systemic circulation via the intestinal lymphatics pathway [19]. These lipophilic molecules and macromolecules are absorbed via lymphatic capillaries, which collect into the mesenteric lymph duct, followed by the thoracic lymph duct, and then drain into systemic circulation at the junction of the left subclavian and left jugular veins [19,20]. Therefore, molecules that are absorbed via the intestinal lymphatic pathway enter systemic circulation without passing through the liver. This alternative absorptive pathway may be of particular importance in drug delivery and may serve as a novel drug delivery approach to minimize the first-pass effect while increasing lymphatic exposure and ultimately improving overall systemic drug exposure [21]. Lipophilic drugs with LogP > 5 and solubility of >50 mg per g in long-chain triglyceride will likely have preferential absorption towards lymphatics owing to their ability to incorporate with intestinal lipoproteins [19]. If the drug of interest does not meet these criteria, it is also possible to alter the physicochemical properties of a small drug molecule by chemically modifying its lipophilicity, utilizing a lipid-based drug delivery system or designing a lipophilic prodrug where the parent drug is chemically conjugated to a lipophilic moiety via a linker that can be easily cleaved in vivo [19,20,21,22]. In this study, we utilized a novel Dox–quercetin conjugate where quercetin is designed to act as a lymphatically targeted carrier and may facilitate the intestinal transport of Dox into systemic circulation after oral administration and may also affect its disposition as well as overall systemic exposure after intravenous administration.

In the light of the studies discussed above and our promising DoxQ observations in vitro, this study was conducted to investigate the feasibility of utilizing the antioxidant quercetin as a lymphatically targeted carrier for Dox with the potential to improve its disposition, oral bioavailability, and tolerability in vivo. We hypothesize that the presence of quercetin in DoxQ, intact or when released from the conjugate, will act as a carrier to transport Dox into lymphatics, at least partially, thus bypassing systemic circulation and increasing the overall bioavailability of Dox. The release of quercetin from DoxQ will likely have a beneficial effect and limit the cardiotoxic and renal side effects of doxorubicin. In addition, the synthesis and change in physicochemical properties of DoxQ may alter its pharmacokinetics and metabolism; the release of quercetin from DoxQ or DoxQ intact may also have effects on CYP3A4 and P–gp, which could further augment the disposition and bioavailability of Dox in vivo. Here, the acute in vivo disposition, safety, and lymphatic uptake of DoxQ are characterized for the first time. The pharmacokinetics, toxicodynamics, and intestinal lymphatic absorption of DoxQ in comparison to free Dox are examined in a rat model. Our results demonstrate that DoxQ improves the disposition of Dox and its oral bioavailability and safety, and is partially transported via lymphatics.

## 2. Materials and Methods

### 2.1. Chemicals and Reagents

Doxorubicin, duanorubicin, cycloheximide, PEG-400, and DMSO were purchased from Sigma (St. Louis, MO, USA). Analytical grade formic acid and HPLC grade acetonitrile were purchased from Fisher Scientific (Ottawa, ON, Canada). Ultrapure water from a Milli-Q^®^ system (Millipore, Billerica, MA, USA) was used for the mobile phase. HPLC columns, vials, inserts, and 0.2 um nylon filter membranes were purchased from Phenomenex^®^ (Torrance, CA, USA). Silastic^®^ laboratory tubing was purchased from the Dow Corning Corporation (Midland, MI, USA). Intramedic^®^ polyethylene tubing was purchased from Becton Dickinson Primary Care Diagnostics, Becton Dickinson and Company (Sparks, MD, USA). Monoject^®^ 23 gauge (0.6 × 25 mm) polypropylene hub hypodermic needles were purchased from Sherwood Medical (St. Louis, MO, USA). Synthetic absorbable surgical sutures were purchased from Wilburn Medical US (Kernesville, NC, USA). Sterile heparin/50% dextrose catheter lock solution and blunt needles were obtained from SAI Infusion Technologies, Strategic Applications (Lake Villa, IL, USA).

### 2.2. Synthesis of the DoxQ Conjugate

DoxQ was synthesized by conjugating Dox to quercetin via a glycine linker, as previously described [14].

### 2.3. Physicochemical Properties

LogP and LogS values of DoxQ were predicted using an online computer software (VCCLAB, Virtual Computational Chemistry Laboratory) [23,24]. pKa, logP, logD at pH 7.4, intrinsic solubility, and solubility at pH 7.4 were calculated using MarvinSketch v. 17.2.20.0 (ChemAxon Ltd., Cambridge, MA, USA), pKa and logP were calculated using GastroPlus v. 9.0.0007 (Simulations Plus, Inc., Lancaster, CA, USA). Portions of these results were generated by GastroPlus™ software (Version 8.0) provided by Simulations Plus, Inc. (Lancaster, CA, USA). The melting point of DoxQ was experimentally determined by MEL-TEMPII melting point apparatus from Laboratory Devices (Holliston, MA, USA).

### 2.4. Analytical System and Conditions 

The analytical method described in [25] was adapted with some modifications. The HPLC system used was a Shimadzu LC-2010A (Kyoto, Japan) with Fluorescence RF-535 detector at 470/560 nm (excitation/emission) wavelengths. Separation was achieved using C18 Phenomenex Kintex^®^ (Torrance, CA, USA) column (250 μm, 250 × 4.6 mm) for serum and lymph samples or (2.6 μm, 100 × 4.60 mm) joined to (250 μm, 250 × 4.6 mm) for urine samples. The mobile phase was prepared by mixing acetonitrile with 0.1% formic acid in water (35:65, *v*/*v*), which was filtered through 0.2 μm nylon filter and degassed under reduced pressure prior to use. The separation was carried out isocratically at ambient temperature (22 ± 1 °C) with a flow rate of 0.6 mL/min. Shimadzu EZStart (Version 7.4) software was used for data collection and integration. On the day of the analysis, samples were prepared and injected into the HPLC system.

#### 2.4.1. Preparation of Standard Solutions

Stock solutions of Dox (1 mg/mL) and the internal standard (IS) duanorubicin (1 mg/mL) were prepared in methanol, protected from light and stored at −20 °C between uses for no longer than one week. Using the stock solutions of Dox, calibration standards in serum, urine, and lymph were freshly prepared by sequential dilution with blank rat serum, urine, and lymph. A series of concentrations were obtained, particularly 0.1, 0.5, 1.0, 10.0 and 100 μg/mL.

Stock solutions of intact DoxQ (10 mg/mL) were freshly prepared in DMSO and protected from light. Calibration standards of DoxQ in serum and urine were prepared by serial dilution with blank rat serum or urine to yield concentrations of 1, 10, 20, and 100 μg/mL. The final concentration of DMSO in serum and urine spiked standards did not exceed 1%.

#### 2.4.2. Calibration Curves

Calibration curves of Dox and DoxQ were obtained by plotting the peak area ratio of Dox or DoxQ to the internal standard (duanorubicin) versus calibration standards concentration of Dox or DoxQ through the unweighted least squares linear regression.

### 2.5. Animals and Surgical Procedures

Male Sprague–Dawley rats (250–300 g) were obtained from Charles River Labs (Montreal, QC, Canada) and given food (Purina Rat Chow 5001) and water ad libitum in the animal facility for at least three days before use. Rats were housed in temperature-controlled rooms with a 12 h light/dark cycle. The animal ethics protocol was revised and approved by the Bannatyne Campus Animal Care Committee at the University of Manitoba, (protocol #16-004, approved 29 March 2016).

### 2.6. Pharmacokinetic Study

Eight surgically modified, with exposed jugular vein catheterization (polyurethane–silastic blended catheter), adult male Sprague–Dawley rats (average weight: 250 g) were purchased from Charles River Laboratories (Saint-Constant, QC, Canada). The cannula was flushed daily with a sterile heparin/50% dextrose catheter lock solution to maintain the patency of the cannula, as advised in the technical sheet supplied with the animals from Charles River. Each animal was placed in a separate metabolic cage overnight and fasted for 12 h before dosing. On the day of experiment, the animals were dosed either intravenously or orally with Dox (10 mg/kg) or equimolar DoxQ (*n* = 4 for each treatment group). Both Dox and DoxQ were freshly reconstituted in 3% DMSO and 97% PEG-400 prior to dosing. Animals received water ad libitum pre- and post-dosing, and food (Purina Rat Chow 5001) was provided 2 h post-dosing. Doses were selected based on previous use in similar pharmacokinetic studies [13,15] and sensitivity of analytical instrumentation. Serial blood samples (0.30 mL) were collected at 0, 1 min, 15 min, and 30 min, then 1, 2, 4, 6, 12, 24, 48 and 72 h after IV administration. The same blood collection time points were applied following oral administration except for 1 min. At 72 h after administration, the animals were euthanized and exsanguinated. Immediately after all the blood collection time points (except the terminal point); the cannula was flushed with the same volume of 0.9% saline to replenish the collected blood volume. The dead volume of the cannula was filled with a small volume (~0.15 mL) of heparinized lock solution after each blood draw to maintain the patency of the cannula. The samples were collected into regular polypropylene microcentrifuge tubes, centrifuged at 15,000 rpm for 5 min (Beckman Microfuge centrifuge, Beckman Coulter Inc., Fullerton, CA, USA), and the serum collected and stored at −20 °C until further sample preparation for HPLC analysis. Urine samples were also collected at 0, 2, 6, 12, 24, 48 and 72 h following Dox or DoxQ administration. The exact urine volume of each sample was recorded then stored at −20 °C until further sample preparation for HPLC analysis.

### 2.7. Intestinal Lymphatic Drug Delivery

The intestinal transport of DoxQ via lymphatics was examined in vivo by two methods. In the first method, mesenteric lymph cannulated rat model was used to directly measure the concentrations of Dox in the lymph after administration of DoxQ or Dox. In the second method cycloheximide, a chylomicron blocking drug, was administered intraperitoneally prior to oral administration of DoxQ or Dox then concentrations of Dox were measured in serum to indirectly assess lymphatic transport.

#### 2.7.1. Mesenteric Lymph Cannulation Surgery

Six male Sprague–Dawley rats (~300 g) were obtained from Charles River Labs (Montreal, QC, Canada) and given food (Purina Rat Chow 5001) and water ad libitum in the animal facility for at least three days before use. On the day of surgical operation, rats were anesthetized by isoflurane and the abdominal hair was shaved. Rats were maintained under inhaled anesthesia on a warm surgical table. A ~2.5 cm abdominal midline skin incision was made and extended through the musculature using blunt dissection beginning the incision at a point just above the xyphoid cartilage and proceeding distally. The intestine and liver were retracted using surgical retractors to locate the superior mesenteric lymph duct, which is filled with opaque white chyle. The lymph duct was isolated from the surrounding connective tissue and a small incision was made with a bent 23 G needle in the ventral wall of the lymph. A catheter was inserted through the incision and secured by placing a small cellulose patch with a drop of Vetbond^TM^ over the point of insertion into the lymph duct. When a gradual and continuous flow of lymph was observed, an initial lymph sample was collected into a normal microtube. A single dose (10 mg/kg) of DoxQ (*n* = 3) or Dox (*n* = 3) was administered by oral gavage while the rat was under anesthesia. Thereafter, lymph samples were collected over one hour after dosing. The animals were euthanized after the last lymph sample collection.

#### 2.7.2. Lymph Blockage by Cycloheximide

Cycloheximide (3 mg/kg) was administered intraperitoneally (IP) to jugular vein cannulated male Sprague–Dawley rats (~250 g) (*n* = 4) 1.5 h prior to oral administration of DoxQ to block the formation of chylomicrons in lymph [26,27,28,29,30,31,32,33,34,35]. DoxQ was then administered orally (10 mg/kg). Blood samples were collected at 0 h, 15 min, 30 min, 1 h, 2 h, 6 h, 12 h, 24 h and 48 h. The animals were euthanized after the last blood sample collection.

### 2.8. Treatment of Biological Samples for Analysis

#### 2.8.1. Serum and Lymph Sample Preparation

To a 100 μL serum or lymph sample (except 0 h), 10 μM of the internal standard (duanorubicin) was added then vortexed for 30 s (Vortex Genie–2, VWR Scientific, West Chester, PA, USA). One milliliter of cold HPLC grade acetonitrile (pre-stored at −20 °C) was added to the precipitate proteins, vortexed for 2 min (Vortex Genie–2, VWR Scientific, West Chester, PA, USA), and centrifuged at 15,000 rpm for 5 min; the supernatant was transferred to new, labeled 2 mL centrifuge tubes. The samples were evaporated to dryness using a Savant SPD1010 SpeedVac Concentrator (Thermo Fisher Scientific, Inc., Asheville, NC, USA). The residue was reconstituted with 100 μL of mobile phase, vortexed for 1 min, and centrifuged at 15,000 rpm for 5 min; the supernatant was transferred to HPLC vials and 100 μL were injected into the HPLC system.

#### 2.8.2. Urine Sample Preparation

Two hundred microliters of urine and 10 μM of the internal standard were combined and vortexed for 30 s. The proteins present in the urine samples were precipitated using 1.6 mL cold HPLC-grade acetonitrile (pre-stored at −20 °C), vortexed for 2 min, and centrifuged at 15,000 rpm for 15 min. The supernatant was transferred to new, labeled 2-mL centrifuge tubes. The samples were evaporated to dryness using SpeedVac. The residue was reconstituted with 200 μL of mobile phase, vortexed for 1 min, and centrifuged at 15,000 rpm for 15 min. The supernatant was transferred to HPLC vials and vortexed, and 100 μL was injected into the HPLC system.

### 2.9. Pharmacokinetic Analysis

Pharmacokinetic analysis was performed using data from individual rats, and the mean and standard error of the mean (SEM) were calculated for each group. The elimination rate constant (*k_el_*) was estimated by linear regression of the serum concentrations in the log-linear terminal phase. Non-compartmental modeling of the serum concentration versus time data points was performed using Phoenix^®^ WinNonlin^®^ software (Version 6.3) (Pharsight Corporation, Mountain View, CA, USA) to calculate the pharmacokinetic parameters in the terminal phase, namely mean residence time (MRT), total clearance (*CL*_tot_), and volume of distribution (*V*_ss)_. The initial maximum serum concentration (*C*_0_) was calculated by back extrapolation using WinNonlin software. Based on the cumulative urinary excretion data, the fraction excreted in urine (f_e_ by dividing the total cumulative amount excreted in urine (ΣXu) by the dose), renal clearance (*CL*_renal_ by multiplying f_e_ by *CL*_tot_), and hepatic clearance (*CL*_hepatic_ by subtracting *CL*_renal_ from *CL*_tot_, assuming that hepatic clearance is equivalent to non-renal clearance) were calculated. The fraction of a dose converted to a specific metabolite (*F*_m_) was calculated using the following equation: *F*_m_ = AUC(_m,D_)/AUC(_m_), where AUC(_m_,_D_) is the AUC of the metabolite after IV or PO administration of its precursor (Dox after DoxQ) and AUC(_m_) is the AUC of the metabolite after IV administration of an equimolar dose of the preformed metabolite (Dox after Dox) [36,37].

### 2.10. Assessment of Cardiac Toxicity of DoxQ and Dox

The cardiac toxicity was assessed after a single IV dose of Dox or DoxQ utilizing a rat cardiac Troponin-I (cTnI) ultra-sensitive ELISA kit from Life Diagnostics, Inc. (West Chester, PA, USA). Blood samples from pharmacokinetic studies were collected at 0, 12, 24, and 48 h from the jugular vein after a single 10 mg/kg IV dose of Dox (*n* = 4) or an equimolar dose of DoxQ (*n* = 4). Samples were centrifuged to obtain the serum and stored at −20 °C in a freezer until analysis. On the day of the analysis, cTnI concentrations were measured in serum samples following the manufacturer’s instructions. The area under the effect curve (AUEC) was calculated for cTnI concentrations at 12–48 h post-dosing using the trapezoidal rule [38,39] by WinNonlin^®^ software. 

### 2.11. Assessment of Renal Toxicity of Dox and DoxQ

#### 2.11.1. Urinary Output

The urinary output of rats over 24 h was monitored before and after administration of a single IV dose of Dox (10 mg/kg) or equimolar DoxQ to assess potential renal toxicity. Acute renal toxicity induced by Dox and other drugs may result in a reduction in the total urinary output [40,41,42]. The total urine volume excreted over 24 h post-dosing was compared to the total urine volume excreted over 24 h pre-dosing.

#### 2.11.2. β-*N*-Acetylglucosaminidase (NAG)

The potential renal toxicity of Dox and DoxQ was determined by measuring β-*N*-acetylglucosaminidase (NAG), a marker of ongoing renal damage, in rat urine [8,43]. Urine samples from pharmacokinetics experiments were collected from metabolic cages at 0 h, 12 h, 24 h, and 48 h and stored at −20 °C until analysis. Concentrations of NAG urine samples were measured using an assay kit from ALPCO Diagnostics (Salem, NH, USA, cat. No. 73-1290050) on a Medica EasyRA automated clinical chemistry analyzer (Medica Corporation, Bedford, MA, USA) [44,45].

### 2.12. Statistical Analysis

Compiled data were presented as mean and standard error of the mean (mean ± SEM). Where possible, the data were analyzed for statistical significance using SigmaPlot software (v. 13.0, Systat Software, Inc., San Jose, CA, USA). Student’s *t*-test was employed for unpaired samples to compare means between two groups, while one-way ANOVA was employed to compare the means of three or more groups, with subsequent *t*-tests between groups if necessary; a value of *p* < 0.05 was considered statistically significant.

## 3. Results

### 3.1. Physicochemical Properties

As DoxQ is a chemical derivative of Dox, the change in the chemical structure of Dox will likely alter the physiochemical properties of the parent drug, which may affect its disposition into biological fluids and pharmacokinetic profile. Therefore, exploring the physicochemical properties of DoxQ in comparison to Dox provides insight into the differences in their dispositions and pharmacokinetics. Computer software, namely VCCLAB [23,24], MarvinSketch, and GastroPlus, were used to predict the physicochemical properties of DoxQ and Dox (Table 1). The estimated partition coefficient (LogP) value of DoxQ (2.6–3.8) was 3–5-fold higher than Dox (logP 0.49–1.3), suggesting the higher lipophilicity of DoxQ. The distribution coefficient at pH 7.4 (LogD_7.4_), which takes into account the ionizable groups at specific pH, of DoxQ was 25-fold higher than Dox (0.097) and may be a better predictor of lipophilicity. The predicted LogP and LogD_7.4_ values of DoxQ are in agreement with the low predicted solubility (0.006 mg/mL) of DoxQ compared to Dox (0.243 mg/mL) at physiological pH and higher logS values of DoxQ. The predicted pKa values of DoxQ were also different than those of Dox. Furthermore, the experimentally determined melting point of DoxQ was 175 °C compared to 242 °C. The difference in the predicted pKa values of DoxQ versus Dox as well as other physicochemical properties described above indicate that DoxQ is distinct from Dox and exhibits unique physicochemical properties.

### 3.2. HPLC Analysis of Dox

**Figure 1:**
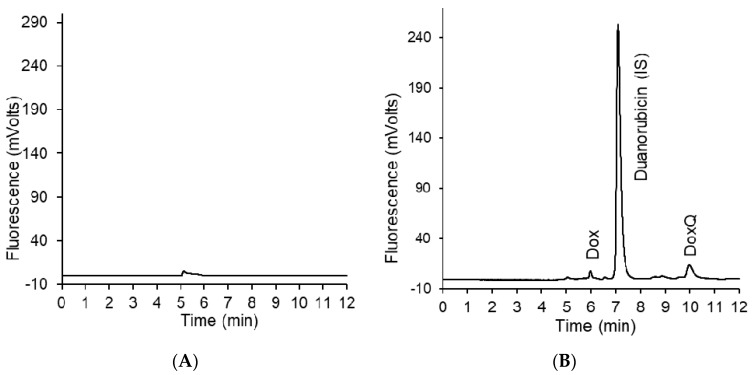
(**A**) Representative chromatogram of blank serum; (**B**) representative chromatogram of Dox, DoxQ, and the internal standard duanorubicin after 30 min of DoxQ IV dosing.

Optimal separation of Dox, DoxQ, and duanorubicin (IS) in serum, urine, and lymph was achieved with a mobile phase composed of acetonitrile with 0.1% formic acid in water 35:65, *v*/*v* and a flow rate of 0.6 mL/min on a C18 Phenomenex Kintex^®^ (Torrance, CA, USA) column. Chromatograms were free of any interfering peaks co-eluted with peaks of interest (Figure 1). Calibration curves of Dox in serum and lymph were linear over the range of 0.05–100 μg/mL in serum and lymph and 0.1–100 μg/mL for urine, with excellent linearity (*r*^2^ > 0.99) in all three matrices. Calibration curves of DoxQ in serum and urine were linear over the range of 1–100 μg/mL (*r*^2^ > 0.99). The observed maximum serum concentration (C_max_) of both Dox and DoxQ at 1 min post-dosing was within the linear range. The limit of quantification (LOQ) was 0.05 μg/mL and 1 μg/mL for Dox and DoxQ intact, respectively.

### 3.3. Pharmacokinetics of Dox and DoxQ

#### 3.3.1. IV Administration

**Figure 2:**
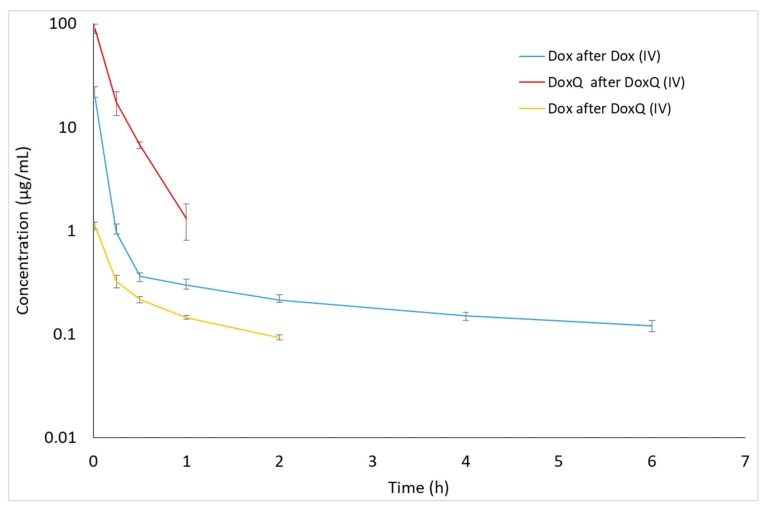
Concentrations of Dox and DoxQ intact after IV administration of Dox (10 mg/kg, *n* = 4 mean ± SEM) or DoxQ (equimolar dose, *n* = 3 mean ± SEM) in rat serum.

**Figure 3:**
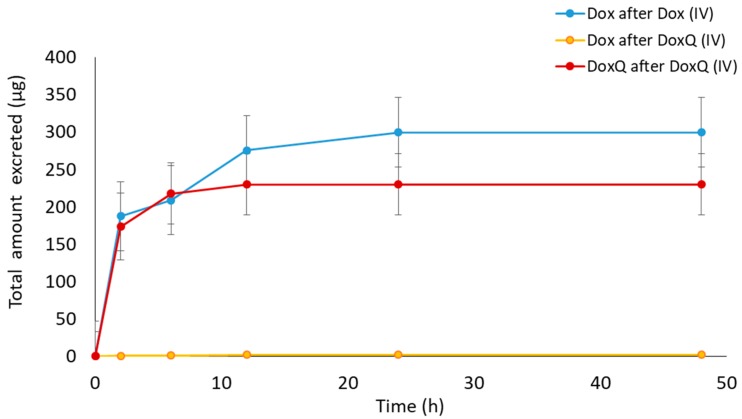
Cumulative amounts of Dox and DoxQ intact excreted unchanged in the urine after IV administration of Dox (10 mg/kg; *n* = 3 mean ± SEM) and equimolar DoxQ (*n* = 4 mean ± SEM) during the 48 h post-dosing.

The disposition profiles of Dox and DoxQ intact in serum and urine following a single IV dose of Dox and an equimolar dose of DoxQ were examined (Figure 2 and Figure 3). The serum concentration–time profile of IV DoxQ showed a rapid decline over the first 30 min and was quantifiable up to 1 h post-dosing. The concentrations of Dox after Dox were quantifiable up to 6 h post-dosing (Figure 2), with a maximum serum concentration (C_0_) of Dox after Dox of ~25 μg/mL. The disposition profile of Dox after Dox, as well as its pharmacokinetic parameters, are consistent with the literature [13,15,25]. Following IV administration of DoxQ, both DoxQ intact and Dox were detected with maximum serum concentration (C_0_) of intact DoxQ of ~108 μg/mL and ~1 μg/mL for free Dox (Table 2). Concentrations of intact DoxQ demonstrated a rapid decline over one hour, while concentrations of Dox after DoxQ dosing showed a slower decline and were quantifiable up to 2 h post-dosing. Notably, the maximum serum concentration (C_0_) of intact DoxQ after equimolar IV DoxQ was 4–5-fold higher than C_max_ of Dox after IV Dox. The area under the concentration–time curve (AUC) of intact DoxQ (18.6 ± 1.98 μg * h/mL) was also 5-fold higher than that of Dox (3.97 ± 0.71 μg * h/mL), demonstrating higher systemic exposure to DoxQ. The volume of distribution V_ss_ of Dox was ~80-fold higher than that of DoxQ, suggesting significantly greater tissue distribution of Dox. The fraction of DoxQ metabolized into Dox was ~12%.

In the urine, both DoxQ intact and Dox were detected after DoxQ IV dosing. Likewise, Dox was excreted unchanged after Dox IV dosing (Figure 3). The total cumulative urinary excretion plots demonstrate that DoxQ is predominantly excreted as intact DoxQ and, to a much lower extent, as free Dox after DoxQ dosing. The total cumulative amount of free Dox excreted unchanged was much higher after Dox dosing than after DoxQ. The fraction of the dose excreted unchanged in the urine (f_e_) of DoxQ and Dox were 4.32 ± 1.005 and 10.73 ± 3.14, respectively, indicating that both drugs are mainly eliminated by non-renal routes.

#### 3.3.2. Oral Administration

**Figure 4:**
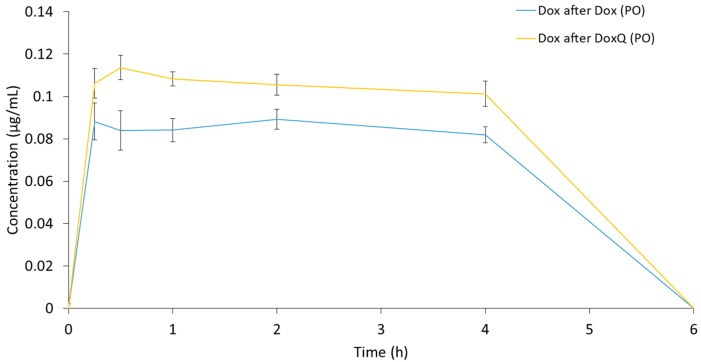
Concentrations of Dox after PO administration of Dox (10 mg/kg) and equimolar DoxQ in rat serum over 6 h. (*n* = 3 mean ± SEM for Dox, *n* = 4 mean ± SEM for DoxQ).

Following oral administration of DoxQ only the metabolite Dox was detected in serum as opposed to both DoxQ intact and Dox after IV administration (Figure 4). Following oral administration of Dox, Dox was also detected in serum. The serum concentration time plots demonstrate that concentrations of Dox after DoxQ were higher than after Dox at all time points, with resultant higher calculated AUC_last_ values of Dox after DoxQ than Dox after Dox when each was orally administered (Table 3). Bioavailability of Dox after Dox was ~8.5%, while the fraction of DoxQ metabolized into Dox was ~10.3%.

### 3.4. Intestinal Lymphatic Drug Delivery

#### 3.4.1. Mesenteric Lymph Duct Cannulation

**Figure 5:**
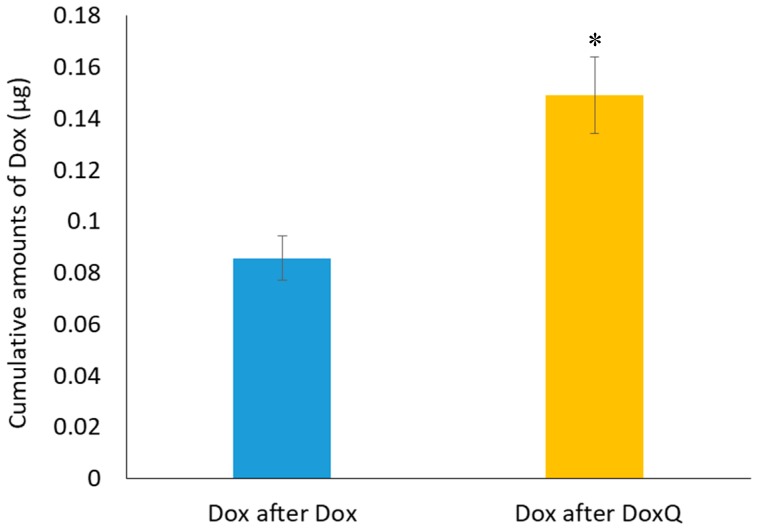
Cumulative amounts of Dox in mesenteric lymph fluid over one hour after oral administration of Dox (10 mg/kg) and equimolar DoxQ (*n* = 3, mean ± SEM). * *p* < 0.05 Dox after DoxQ versus Dox after Dox.

The mesenteric lymph duct cannulation rat model is commonly used as to directly examine the transport of drugs after oral administration because it enables the collection of lymphatic fluids as it flows from the intestine [20,35]. The intestinal lymphatic transport of Dox after oral administration DoxQ and Dox was investigated in a mesenteric lymph duct cannulated model to assess whether the presence of quercetin facilitates lymphatic transport of Dox. Following oral DoxQ or Dox dosing, lymph samples were collected up to one hour post-dosing and concentrations of Dox were measured by HPLC. The cumulative amount of Dox in mesenteric lymph fluid after oral DoxQ were two-fold higher than after Dox (Figure 5), suggesting that quercetin in DoxQ, intact or when released, increased the intestinal delivery of Dox into lymphatics.

#### 3.4.2. Lymph Blockage by Cycloheximide

Intestinal lymphatic delivery of Dox was also examined indirectly in the cycloheximide treated rat model. Lymph blockage was achieved by pre-administration of cycloheximide 1.5 h prior to oral administration of DoxQ. A 3 mg/kg intraperitoneal dose of cycloheximide was chosen based on previous studies published in the literature [26,27,28,29,30,31,32,33,34,35]. Likewise, a 1.5-h time delay prior to oral DoxQ dosing was chosen to achieve maximum lymph blockage [29].

**Figure 6:**
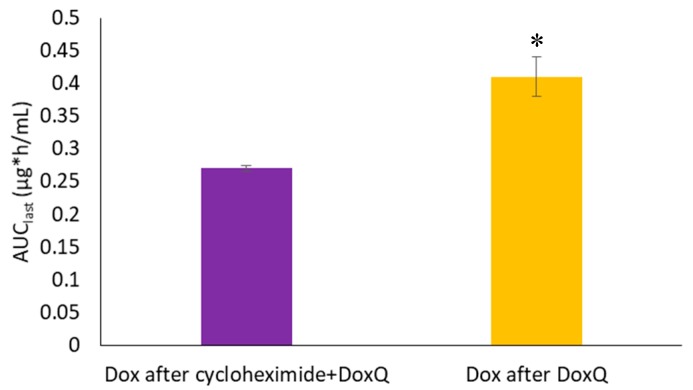
Systemic exposure (AUC_last_) of Dox after PO administration of DoxQ alone or after cycloheximide IP followed by DoxQ PO in rat serum (*n* = 4 mean ± SEM). * *p* < 0.05 Dox after DoxQ versus Dox after cycloheximide + DoxQ.

Figure 6 demonstrates that pre-administration of cycloheximide prior to oral DoxQ reduced the systemic exposure of Dox compared to DoxQ administered alone. Given that quercetin is naturally transported via intestinal lymphatics and could act as a carrier for Dox’s (Dox in DoxQ) lymphatic transport, blockage of the intestinal lymphatic pathway may reduce systemic exposure. The results suggest that quercetin in DoxQ, intact or when released from the conjugate, facilitates intestinal lymphatic transport of Dox, and that blocking the lymphatic pathway resulted in lower levels of circulating Dox. 

### 3.5. Cardiotoxicity of Dox and Dox

**Figure 7:**
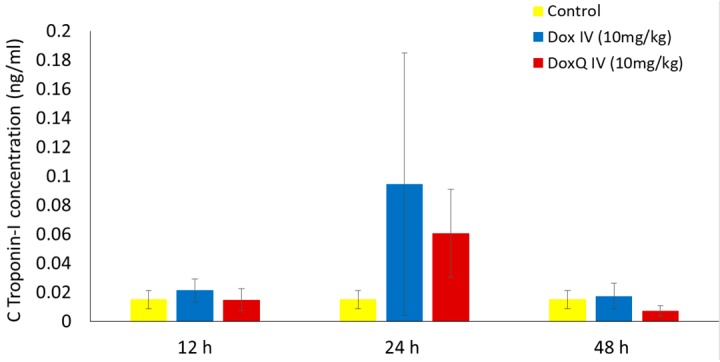
cTnI concentrations after IV (10 mg/kg) administration of Dox and equimolar DoxQ (*n* = 4 mean ± SEM). Data were not statistically significant.

**Figure 8:**
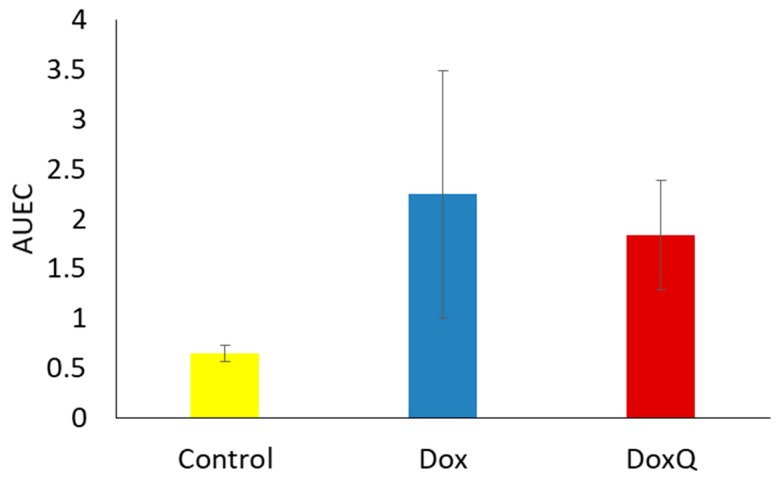
AUEC of cTnI concentrations 12-48 h after IV (10 mg/kg) administration of Dox and equimolar DoxQ (*n* = 4 mean ± SEM). Data were not statistically significant.

The clinical use of Dox is limited by its dose-related cardiotoxicity, which can result in cardiac muscle injury. The extent of myocardial injury can be assessed by measuring the levels of cardiac troponins in blood. Cardiac troponins are highly sensitive and specific biomarkers of cardiac muscle damage, induced by chemotherapeutics as well as other pathological conditions [47]. cTnI is commonly used as an early marker of cardiotoxicity induced by Dox [8,48] as it is released within 2–3 h of myocardial injury and peaks at 24 h [49,50]. Based on the reported peak troponin concentrations following myocardium injury, levels of cTnI were measured in serum samples from pharmacokinetic study at 12, 24, and 48 h after a single acute IV dose (10 mg/kg) of Dox or equimolar DoxQ utilizing an ELISA kit. Figure 7 illustrates that the concentrations of cTnI at 12, 24, and 48 h post-IV-dosing of DoxQ were lower than after Dox dosing, though this did not reach statistical significance and thus the cardiac toxicity induced by DoxQ and Dox was not different using this biomarker. Although the calculated area under the effect curve (AUEC) of cTnI concentrations at 12–48 h post-DoxQ-dosing (Figure 8) was lower than the AUEC after Dox, it did not result in cardioprotective effects of DoxQ as there were no statistical differences between the treatment groups.

### 3.6. Renal Toxicity of Dox and DoxQ

#### 3.6.1. Urinary Output over 24 h

**Figure 9:**
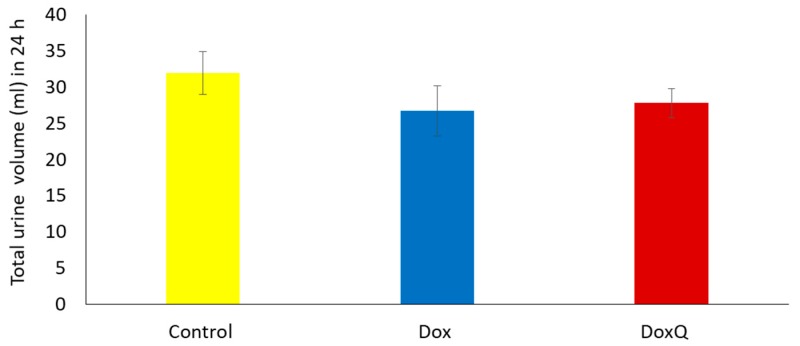
Average total urine volume 24 h post IV Dox (10 mg/Kg) and equimolar DoxQ compared to control untreated. Dox (*n* = 3 Mean ± SEM), DoxQ (*n* = 4 Mean ± SEM), control (*n* = 7 Mean ± SEM). Data were not statistically significant.

Dox can also induce renal toxicity, which could be manifested as reduced urinary output. Thus, the effect of DoxQ on the total urinary output of rats over 24 h was examined in comparison to rats treated with Dox after a single acute IV dose. Figure 9 shows that there was no significant difference in the total urine volume over 24 h in rats treated with Dox or an equimolar dose of DoxQ.

#### 3.6.2. β-N-Acetylglucosaminidase (NAG)

**Figure 10:**
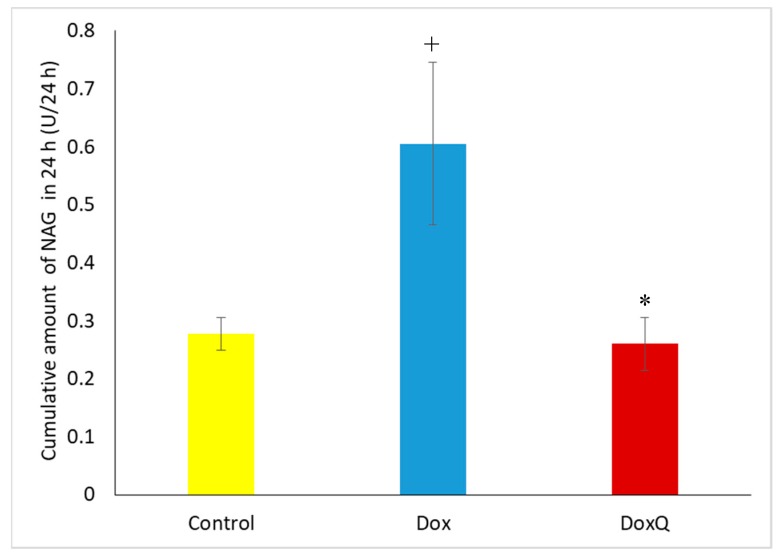
Total amount of NAG excreted in urine after IV administration of Dox (10 mg/kg) mean ± SEM) and equimolar DoxQ compared to control untreated (*n* = 4, mean ± SEM). + *p* < 0.05 Dox versus control ,* *p* < 0.05 DoxQ versus Dox.

The potential renal toxicity of Dox and DoxQ was determined by measuring β-*N*-acetylglucosaminidase (NAG), a lysosomal enzyme found in large concentrations in kidney tubules and a sensitive and early marker of renal damage [8,51,52]. Urine samples from pharmacokinetic studies after a single IV dose of DoxQ or Dox collected at 0, 2, 6, 12, 24 and 48 h were analyzed on a Medica easy RA analyzer for NAG concentrations. Cumulative amounts of NAG in 24 h after DoxQ dosing were lower than after Dox (Figure 10), suggesting lower renal toxicity induced after DoxQ administration compared to Dox.

## 4. Discussion

Dox is an anthracycline antibiotic widely used in cancer chemotherapy; however, its dose-dependent toxicity limits its clinical use. The purpose of this study was to investigate the feasibility of utilizing a Dox-bound derivative with the lymphatically absorbed antioxidant quercetin as a proof of concept linker, delivering Dox and Dox–quercetin to the systemic circulation via intestinal lymphatics after oral dosing. In addition, the flavonoid–Dox conjugate may lead to sustained release of the anti-cancer agent after IV dosing, resulting in lower peak serum concentration, which may be associated with the dose-limiting cardiotoxicity of the drug. Furthermore, the protective antioxidant effects of quercetin in DoxQ, when intact or when released, may further limit the cardiac and renal toxicity induced by Dox.

Various liposome-encapsulated formulations of Dox are currently available in human studies with the advantage of reduced acute cardiotoxicity compared to IV Dox and improved pharmacokinetics [53]. However, pegylated liposomal Dox is associated with an additional side effect of palmar plantar erythrodysesthesia [7]. In this study, we sought to examine the performance of a controlled-release Dox–quercetin conjugate with reduced side effects in vitro, which may have better tolerability compared to conventional Dox–HCl and liposomal Dox treatments.

The Dox–quercertin conjugate was synthesized using a glycine linker, resulting in a new derivative with increased lipophilicity and improved in vitro pharmacological activities. Our previous in vitro study demonstrated a controlled release of both Dox and quercetin released from DoxQ over four days [14]. Furthermore, DoxQ was less cardiotoxic than Dox to both rat and human cardiomyocytes and the mechanism of cardioprotection involved a reduction in the levels of ROS and oxidative stress markers as well as inhibitory effects on the expression and catalytic activity of CYP1B1. Additionally, DoxQ mitigated the therapeutic barriers contributing to the low oral bioavailability of Dox as it inhibited CYP3A4 and demonstrated higher cellular uptake by P–gp-positive cells (MDCK–MDR) in vitro.

In this study, our intestinal lymphatic delivery strategy was applied to the DoxQ delivery system, increasing the serum concentrations of Dox at all time points, with an overall increase in AUC_last_ of Dox after oral DoxQ administration compared to after Dox, reflecting an overall increase in the systemic exposure of Dox. In addition, only free Dox was released from oral DoxQ and detected analytically, thus it was analyzed for systemic exposure. Given that DoxQ was not detected as the intact conjugate after oral dosing of DoxQ, calculating the F of DoxQ intact was not possible; however, it was appropriate to calculate the fraction of DoxQ metabolized to Dox, as described in Section 2.9. Therefore, the extent of systemic exposure of Dox after oral Dox and DoxQ was assessed by comparing the AUCs of Dox (Table 3).

After IV administration DoxQ was detected intact, which we have shown to be a pharmacologically active form as it retained anti-cancer activity in a triple-negative murine breast cancer cell line [14]. The actual total concentration of DoxQ intact in the serum after an equimolar dose of Dox was 5-fold higher than the concentration of Dox after Dox, allowing a greater AUC compared to the standard Dox treatment. This could result in a lower dose of DoxQ being required to achieve the same effective serum concentrations. DoxQ injections could conceivably be as effective as Dox with reduced toxicity. With regard to its pharmacokinetics, the difference in the volume of distribution between the IV Dox after Dox group and the IV DoxQ intact group was possibly due to the change in physicochemical properties and inhibition of P–gp by quercetin. Dox with a log P value of 1.3, pKa of 8.4, and a molecular weight of 543.53 g/mol rapidly crosses the lipid membrane and binds to tissues, resulting in a larger V_ss_. On the other hand, DoxQ with a smaller V_ss_ (0.08) indicates that it is should mainly reside in the vascular compartment with much lower affinity to distribute across biological membranes compared to Dox, regardless of its higher lipophilicity (logP 2.60–3.8). This could be due to the large molecular weight of 982 g/mol and the presence of multiple potential ionization sites, which may impede its distribution across biological membranes while intact. Similar effects were observed for clozapine nano-formulation, where tissue distribution of clozapine incorporated in solid lipid nanoparticles was lower than clozapine solution because free clozapine could only be distributed after its release from nanoparticles [54]. Therefore, it is possible that DoxQ intact will distribute to a lower extent than when it is released from the conjugate or when Dox alone is administered. Examination of the total cumulative amounts of Dox and DoxQ excreted unchanged after IV dosing revealed lower cumulative amounts of DoxQ intact after DoxQ than of Dox after Dox. This could be due to the large molecular weight of DoxQ (928.8 g/mole) to be filtered at the glomerulus and also its high lipophilicity (LogP 2.6–3.8, Table 2), as opposed to Dox with a smaller size and lower lipophilicity.

With regard to intestinal lymphatic absorption of DoxQ, our results show that cumulative amounts of Dox following DoxQ oral dosing were twice as high after Dox in a mesenteric lymph cannulated rat model. This observation is likely due to the presence of quercetin in DoxQ, intact or when released, acting as a lymphatically targeted carrier to facilitate the transport of Dox into lymphatics, as quercetin has been reported to be transported via intestinal lymphatics following intragastric or intraduodenal administration [16,17,18]. Additionally, compounds with high lipophilicity, high logP, and large molecular size favor association with chylomicrons in the intestine, facilitating their uptake by lymphatic capillaries into the mesenteric lymph duct [19,21]. The new derivative is more lipophilic (LogP 2.6–3.8) and larger in size (molecular weight 928.8 g/mole) compared to Dox (LogP 1.3, 543.53 g/mol), both of which may in part facilitate DoxQ’s lymphatic intestinal absorption. Furthermore, formulation effects of PEG-400 on the intestinal absorption and pharmacokinetics of DoxQ and even Dox are possible. PEG-400 is often utilized in dosing of rodent species [45,55] and was used as a vehicle in this study for both oral and IV administration of DoxQ and Dox because DoxQ has poor water solubility and reconstitution in 0.9% NaCl is not feasible. The diverse effects of PEG-400 on solubility, permeability, drug metabolizing enzymes, transporters, and gastrointestinal transit time may have influences on the intestinal absorption and systemic exposure of oral drugs [56,57,58].

In spite of the efficacy of Dox chemotherapy, its clinical use is limited due to its dose-limiting cardiac toxicity along with its renal toxicity, caused in part by the generation of oxygen species in the conversion from Dox to semiquinone, yielding very reactive hydroxyl radicals. The free radical may also cause damage to various membrane lipids and other cellular components [59]. Following a large single-dose injection of Dox (10 mg/kg IV), there was an increase in cTnI released from cardiac tissues at the 12, 24, and 48 h time points, consistent with the literature. In parallel, rats that received DoxQ (equimolar dose of Dox) also had an increase in cTnI at each time point. The significant 4–5-fold increase of circulating intact DoxQ as compared to Dox and its overall increase in systemic exposure, as well as the metabolism of DoxQ to Dox, did not result in higher cTnI compared to rats that received Dox as no statistically significant difference in cardiac toxicity was observed between the two treatment groups (Figure 7 and Figure 8). The in vivo cardiac effects of DoxQ in this study are different from our in vitro observations, where DoxQ formulation greatly reduced the cardiac toxicity induced by Dox in both rat and human cardiomyocytes [14]. This observation is also different from a reported study in which the combination of Dox with resveratrol and quercetin polymeric micelles was shown to mitigate Dox-induced cardiotoxicity both in vitro and in vivo [60]. The in vivo cardioprotective effects of this combination strategy were assessed by measuring levels of AST, ALT, and CK in mice and showed a significant reduction in all three biochemical markers as opposed to Dox administered alone. The observations from the later reported study [60] and our in vitro study [14] demonstrate the protective effects of quercetin on Dox-induced cardiotoxicity. The cardioprotective effects reported in [60] were attributed to the synergistic action of resveratrol and quercetin micelles when co-administered with Dox at a ratio of 10:10:1 resveratrol:quercetin:Dox, whereas our DoxQ conjugate is designed to release Dox and quercetin at a ratio of 1:1, which may have not been enough to show cardioprotection in vivo after one dose. Additionally, the use of two antioxidants, namely resveratrol and quercetin, together could have provided a greater ability to scavenge reactive oxygen species and attenuate the cardiotoxicity induced by Dox as opposed to only quercetin in the DoxQ formulation.

Urine analysis following a single acute dose of Dox showed higher cumulative amounts of β-N-acetylglucosaminidase (NAG), a lysosomal enzyme in the epithelial cells of the proximal tubules and a sensitive marker of renal damage, compared to DoxQ. This is likely due to the antioxidant protective effects of quercetin in DoxQ on Dox-induced renal toxicity and is consistent with similar studies reported in the literature [61,62].

DoxQ by injection could have greater benefits over standard dosing regimens in terms of tolerance and potential improved toxicity. Further translational efforts will focus on optimizing dose frequency, completing preclinical proof of concept in chronic studies, and examining other natural lymphatic carriers for oral delivery.

## 5. Conclusions

DoxQ alters the pharmacokinetic disposition of Dox both orally and intravenously and is in part transported through intestinal lymphatics. DoxQ may increase therapeutic safety compared to Dox in a rodent model and further long-term studies are warranted.

## Figures and Tables

**Table 1:** Physicochemical properties of Dox, quercetin, and DoxQ.

Compound	Doxorubicin (Free Base)	Quercetin	DoxQ
Structure	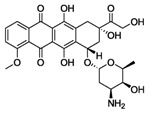	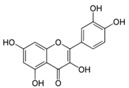	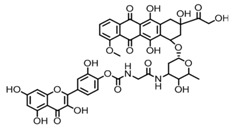
Molecular Weight (g/mol)	543.53	302.238	928.82
Formula	C_27_H_29_NO_11_	C_15_H_10_O_7_	C_45_H_40_N_2_O_20_
pKa (MarvinSketch)	8.00, 9.17, 9.93, 12.67, 13.49, 14.10	6.38, 7.85, 8.63, 10.29, 12.82	6.37, 7.72, 7.94, 8.97, 9.51, 10.21, 12.53, 13.10, 13.57, 14.06, 14.77
pKa (GastroPlus)	6.77, 8.43, 9.5	7.24, 8.15, 9.12, 10.25, 11.35	7.21, 8.08, 8.78, 9.38, 9.91, 10.64, 11.26
pKa (GastroPlus, after fitting solubility)	6.974, 10.08	6.582, 8.15, 10.25, 11.35	7.978, 8.08, 8.78, 9.38, 10.64, 11.26
logP (MarvinSketch)	1.30	1.75	2.60
logP (neutral, GastroPlus)	0.49	1.96	2.61
logP (VCCLAB)	1.3	1.44 ± 0.55	3.8 ± 1.5
logD_7.4_ (MarvinSketch)	0.097	1.00	2.407
Intrinsic solubility (MarvinSketch)	−4.05 logS	−2.49 logS	−6.47 logS
Solubility at pH 7.4 (MarvinSketch)	−3.27 logS	−1.42 logS	−5.34 logS
Solubility at pH 7.4 (MarvinSketch)	0.243 mg/mL	15.15 mg/mL	0.006 mg/mL
logS (VCCLAB)	2.7	2.78	3.43
Melting point (experimental)	242 °C	316.5 °C *	175 °C

* PubChem [46].

**Table 2:** Pharmacokinetics of Dox and DoxQ intact in rat serum after IV administration of Dox (10 mg/kg) and an equimolar dose of DoxQ (mean ± SEM, *n* = 4 unless otherwise stated).

Pharmacokinetic Parameter	Dox Administered	DoxQ Administered	
	Dox	DoxQ ^1^	Dox
C_0_ (μg/mL)	24.7 ± 14.2	108 ± 26.4 *	1.23 ± 0.11 ^+^
k_el_ (h^−1^)	0.16 ± 0.02	4.59 ± 0.78 *	0.75 ± 0.076 ^+^
t_1/2_ (h)	4.69 ± 0.8 ^@^	0.16 ± 0.3 *	0.87 ± 0.07
C_last_ (μg/mL)	0.12 ± 0.03	1.11 ± 0.17 *	0.09 ± 0.01 ^+^
T_last_ (h) ^2^	6	1	2
AUC_last_ (μg * h/mL)	3.97 ± 0.7 ^@^	18.6 ± 1.98 *	0.46 ± 0.04 ^+^
AUC_inf_ (μg * h/mL)	4.79 ± 1.83	NC	0.62 ± 0.03
V_ss_ (L/kg)	6.35 ± 2.11	0.08 ± 0.015	NA
CL_renal_ (L/h/kg) ^1^	0.28 ± 0.84	0.02 ± 0.005	NA
CL_hepatic_ (L/h/kg) ^1^	2.35 ± 0.36	0.51 ± 0.06*	NA
CL_total_ (L/h/kg) ^1^	2.63 ± 0.39	0.53 ± 0.01 *	NA
f_e_ (%)	10.73 ± 3.14	4.32 ± 1.005	NA
f_m_ (%)	NA	NA	11.66 ±0.86

^1^ *n* = 3; ^2^ Median; NC = not calculable because *r*^2^ < 0.8 or AUC% extrapolated > 27%; NA = not applicable; * *p* < 0.05 Dox after Dox versus DoxQ after DoxQ, ^+^ *p* < 0.05 DoxQ after DoxQ versus Dox after DoxQ, ^@^ *p* < 0.05 Dox after Dox versus Dox after DoxQ.

**Table 3:** Pharmacokinetics of Dox after oral administration of 10 mg/kg Dox and equimolar DoxQ in rat serum (mean ± SEM, *n* = 4 unless otherwise stated).

Pharmacokinetic Parameter	Dox Administered	DoxQ Administered	Cycloheximide + DoxQ Administered
	Dox ^1^	Dox	Dox
C_max_ (μg/mL)	0.09 ± 0.01	0.11 ± 0.01 ^+^	0.07 ± 0.001
C_last_ (μg/mL)	0.08 ± 0.01	0.10 ± 0.01	0.07 ± 0.02
T_last_ (h) ^2^	4	4	4
AUC_last_ (μg * h/mL)	0.33 ± 0.04	0.41 ± 0.03 ^+^	0.27 ± 0.005
F_m_ (%)	NA	10.32 ± 0.42 ^+^	6.81 ± 0.14
F (%)	8.57 ± 0.71	NA	NA

^1^ *n* = 3; ^2^ Median; NA = Not applicable; ^+^ *p* < 0.05 Dox after DoxQ versus Dox after Cycloheximide + DoxQ.
